# Weekly, Seasonal, and Festive Period Weight Gain Among Australian Adults

**DOI:** 10.1001/jamanetworkopen.2023.26038

**Published:** 2023-07-27

**Authors:** Carol Maher, Ty Ferguson, Rachel Curtis, Wendy Brown, Dorothea Dumuid, Francois Fraysse, Gilly A. Hendrie, Ben Singh, Adrian Esterman, Timothy Olds

**Affiliations:** 1 UniSA Allied Health and Human Performance, Alliance for Research in Exercise, Nutrition and Activity, University of South Australia, Adelaide, South Australia, Australia; 2 School of Human Movement and Nutrition Sciences, University of Queensland, St Lucia, Brisbane, Australia; 3 Health and Biosecurity, Commonwealth Scientific and Industrial Research Organisation, Adelaide, South Australia, Australia; 4 UniSA Clinical and Health Sciences, Alliance for Research in Exercise, Nutrition and Activity, University of South Australia, Adelaide, South Australia, Australia

## Abstract

**Importance:**

Obesity is a major global health concern. A better understanding of temporal patterns of weight gain will enable the design and implementation of interventions with potential to alter obesity trajectories.

**Objective:**

To describe changes in daily weight across 12 months among Australian adults.

**Design, Setting, and Participants:**

This cohort study conducted between December 1, 2019, and December 31, 2021 in Adelaide, South Australia, involved 375 community-dwelling adults aged 18 to 65 years. Participants wore a fitness tracker and were encouraged to weigh themselves, preferably daily but at least weekly, using a body weight scale. Data were remotely gathered using custom-developed software.

**Exposure:**

Time assessed weekly, seasonally, and at Christmas/New Year and Easter.

**Main Outcomes and Measures:**

Data were visually inspected to assess the overall yearly pattern in weight change. Data were detrended (to remove systematic bias from intraindividual gradual increases or decreases in weight) by calculating a line of best fit for each individual’s annual weight change relative to baseline and subtracting this from each participant’s weight data. Multilevel mixed-effects linear regression analysis was used to compare weight across days of the week and seasons and at Christmas/New Year and Easter.

**Results:**

Of 375 participants recruited, 368 (mean [SD] age, 40.2 [5.9] years; 209 [56.8%] female; mean [SD] baseline weight, 84.0 [20.5] kg) provided at least 7 days of weight data for inclusion in analyses. Across the 12-month period, participants gained a median of 0.26% body weight (218 g) (range, −29.4% to 24.0%). Weight fluctuated by approximately 0.3% (252 g) each week, with Mondays and Tuesdays being the heaviest days of the week. Relative to Monday, participants’ weight gradually decreased from Tuesday, although not significantly so (mean [SE] weight change, 0.01% [0.03%]; *P* = .83), to Friday (mean [SE] weight change, −0.18% [0.03%]; *P* < .001) and increased across the weekend to Monday (mean [SE] weight change for Saturday, −0.16% [0.03%]; *P* < .001; mean [SE] weight change for Sunday, −0.10% [0.03%]; *P* < .001). Participants’ weight increased sharply at Christmas/New Year (mean [SE] increase, 0.65% [0.03%]; *z* score, 25.30; *P* < .001) and Easter (mean [SE] weight change, 0.29% [0.02%], *z* score, 11.51; *P* < .001). Overall, participants were heaviest in summer (significantly heavier than in all other seasons), were lightest in autumn (mean [SE] weight change relative to summer, −0.47% [0.07%]; *P* < .001), regained some weight in winter (mean [SE] weight change relative to summer, −0.23% [0.07%]; *P* = .001), and became lighter in spring (mean [SE] weight change relative to summer, −0.27% [0.07%]; *P* < .001).

**Conclusions and Relevance:**

In this cohort study of Australian adults with weekly and yearly patterns in weight gain observed across 12 months, high-risk times for weight gain were Christmas/New Year, weekends, and winter, suggesting that temporally targeted weight gain prevention interventions may be warranted.

## Key Points

**Question:**

How does daily weight change across 12 months in Australian adults?

**Findings:**

In this cohort study of 368 adults, weight fluctuated by 0.3% each week, with participants gaining a median 0.26% body weight in 12 months. Participant weight increased sharply at Christmas/New Year and Easter, was heaviest in summer, and was lightest in autumn.

**Meaning:**

This study found that the Christmas/New Year period, which occurs in summer in Australia, and winter were key periods for weight gain, suggesting that weight gain prevention interventions targeting these times of the year may be warranted.

## Teaser

This cohort study assesses 12-month weight changes by the week, season, and festive period among Australian adults to better understand temporal patterns of weight gain and loss in the southern hemisphere and to inform weight gain prevention interventions worldwide.

## Introduction

Obesity is a major health concern across the globe, increasing the risk for many diseases and conditions, including heart disease, stroke, type 2 diabetes, osteoarthritis, and certain types of cancer.^1^ It is also associated with negative social and psychological impacts, such as low self-esteem, discrimination, and depression.^2^ The prevalence of overweight and obesity continues to increase around the world, and in Australia, 2 of 3 adults are now overweight or obese.^3^ By 2025, annual obesity-related medical costs are estimated to exceed US $1 trillion globally,^4^ and 7% of Australia’s total health burden is attributed to this problem.^3^ Consequently, considerable effort is being made to understand the factors that contribute to weight gain so that they may be targeted in weight gain prevention programs.

Risk factors for obesity are numerous and range from individual genetic, biological, and psychological to external social, economic, and physical environmental factors, all of which influence eating and activity behaviors. Longitudinal studies suggest that Australian adults aged less than 65 years gain up to 0.5 kg^5,6^ and 0.6 cm of waist girth^5^ per year. However, the temporal evolution of this weight gain across the year is currently poorly understood.

Studies of festive periods, and particularly the holiday season (from approximately late November to early January, taking in Christmas and New Year’s Eve in many countries), show that weight gain coincides with cultural celebrations. For example, weight gain in the US during that season is approximately 0.4 to 0.9 kg^7^ and is thought to account for the majority of annual weight gain.^8^ Comparisons of weight change in 3 northern hemisphere countries (US, Germany, and Japan) have also found that weight gain coincides with cultural celebrations; weight gain at Christmas and Easter in Germany is almost double that of the US, and, in Japan, most weight gain occurs during the May Golden Week period.^9^

A study of seasonal changes in weight and health behavior in the Netherlands suggest that weight peaks in winter and hits a trough in summer.^10^ Seasonal differences in energy intake and diet composition have been reported, although they are generally small in magnitude, and the directions of such differences are inconsistent across studies.^11,12^ Regarding activity behaviors, recent reviews have reported seasonal variations in physical activity and sedentary behavior, with more physical activity in summer and more sedentary behavior in winter.^13,14^

Today’s advanced mobile technologies mean population studies can collect comprehensive lifestyle and weight data frequently over long periods and with minimal participant burden (eg, using motion sensors and Bluetooth scales to remotely monitor activity and body weight). Such technologies offer new opportunities to explore the mechanisms underpinning the development of obesity. A better understanding of temporal patterns of weight gain will enable the design and implementation of interventions for when they are needed most, with potential to alter obesity trajectories.

The aim of this study was to describe changes in daily weight across a 12-month period among Australian adults. Specifically, we aimed to (1) examine whether weight varied across the week and according to season; (2) determine whether weight changed in particular festive periods (eg, Christmas/New Year); and (3) describe the patterns of daily weight changes (relative to baseline) among adults who maintained, gained, or lost weight across the 12-month period.

## Methods

### Study Design

Data for this cohort study were obtained from the cohort study Annual Rhythms in Adults’ Lifestyle and Health (ARIA), which collected 13 months of data between December 1, 2019, and December 31, 2021.^15^ The present study obtained daily data from adults on weight and 24-hour movement behaviors. Full details of the study protocol are published elsewhere.^15^

The University of South Australia Human Research Ethics Committee provided ethical approval. The study protocol was prospectively registered with the Australian New Zealand clinical trial registry (ACTRN12619001430123). This project was conducted in accordance with the Declaration of Helsinki. Written informed consent was obtained from all participants. This study followed the Strengthening the Strengthening the Reporting of Observational Studies in Epidemiology (STROBE) guidelines.^16^

### Setting and Participants

In total, 375 community-dwelling adults were recruited from Adelaide, South Australia. Participants included 119 parents of children enrolled in a separate cohort study examining children’s lifestyles across the school and school holiday periods^17^ (cohorts 1 and 2) and 256 parents of primary school-aged children recruited from the general community using a range of recruitment strategies (eg, social media, community notice boards, mainstream media; cohort 3). Participants were recruited in 2 waves. Cohort 1 commenced data collection on December 1, 2019, and cohorts 2 and 3 commenced data collection on December 1, 2020. Participants’ data collection took place across 13 months although the data presented here are for 12 months.

The inclusion criteria for this study were ambulant adults aged 18 to 65 years who were a parent or guardian of a school-age child residing in the greater metropolitan Adelaide area with internet access (either via smartphone or home internet connection) and the ability to read and understand English. Exclusion criteria included being pregnant, having an implanted electronic medical device, or experiencing a life-threatening condition that affected daily lifestyle and health.

Participants received 1 home visit from study personnel prior to study commencement. At that visit, participants were provided with remote monitoring technology (Fitbit Aria body weight scale [Aria 2 or Aria Air scales] and Fitbit Charge 3 fitness tracker; Fitbit Inc) and were instructed in the use of the devices. In addition, participant height was measured objectively. Participants completed a baseline survey providing their demographic characteristics. At study completion, participants were allowed to keep the scale and activity tracker and received an honorarium of A$ 100 (approximately US $65).

### Variables

#### Weight

Participants were encouraged to weigh themselves, preferably daily, first thing in the morning, while wearing minimal clothing and after voiding. Weight data were collected remotely using Fitnesslink software (Portal Australia). This software was purpose-built for this study.

#### Height

Height was objectively measured during the home visit, using a stadiometer (Tanita Leicester Portable Height Measure), and following International Society for the Advancement of Kinanthropometry assessment procedures.^18^ Baseline body mass index (calculated as weight in kilograms divided by height in meters squared)^2^ was categorized as underweight (<18.5), normal (18.5-24.9), overweight (25.0-29.9), and obese (≥30.0).

### Demographic Characteristics

Demographic characteristics included self-reported sex, date of birth, Indigenous status, marital status, country of birth, smoking status, chronic conditions, number of adults and children in the household, and highest educational level.

### Statistical Analysis

Demographic data were analyzed descriptively using means and SDs for continuous data (eg, age) and counts and percentages for categorical data (eg, weight status). To visualize change in daily weight across the year, each participant’s daily weight was normalized by subtracting the starting weight on December 1 (commencement of data collection) and dividing by their mean weight for the year. Daily weight fluctuations were smoothed using a 7-day running mean. To remove systematic bias based on people who gradually lost or gained weight across the year, the data were detrended, as described by Helander.^9^ For this calculation, a line of best fit was derived for each individual to account for each individual’s annual weight change, which was subtracted from their weight data. A graph was then created displaying the percentage weight change across the 12-month period. Visual inspection of the data was used to detect periods of weight gain, with particular attention paid to Christmas/New Year and Easter.

Weight change categories were calculated based on the mean weight in the first 2 weeks of December compared with the mean weight in the last 2 weeks of the following November. Participants in the subgroup that maintained weight were individuals whose 12-month weight change was within 2% of their baseline weight; participants in the subgroup that gained weight were individuals who gained more than 2% of their baseline weight across 12 months; and participants in the subgroup that lost weight were individuals who lost more than 2% of their baseline weight across 12 months.^19^ Separate 12-month weight change graphs were created for the 3 weight change subgroups to visually identify differences in weight change across the 12-month period.

Days of the week (Monday to Sunday) and seasons (using the middle month of each season, ie, summer, January; autumn, April; winter, July; and spring, October) were compared using multilevel mixed-effects linear regression analyses in Stata, version 17 (StataCorp), with statistical significance set at *P* < .05. All analyses were 2 sided. Multilevel modeling with random intercepts was used to adjust for the nonindependence of the data and to account for nesting of repeated measures within individuals, individuals within families, and families within waves. Percentage weight change relative to baseline was the dependent variable, with day of the week and season included as fixed effects (1 included per model). The regression coefficients for day of the week and season were used to identify differences in percentage weight change associated with a change in day or season, respectively.

## Results

Most participants (368 of 375 [98.1%]) provided at least 7 days of weight data for inclusion in the analytic data set. The 368 participants whose data were analyzed (mean [SD] age, 40.2 [5.9] years; range, 27-65 years); 209 [56.8%] female; 159 [43.2%] male) provided a mean (SD) of 268 (99) days (range, 7 to 363 days) of weight data. At baseline, their mean (SD) weight was 84.0 (20.5) kg. Approximately one-third of participants was within the range of each of 3 body mass index categories: normal, overweight, and obese. Most participants had some form of education after high school, fewer than 1 of 10 smoked, and more than half reported 1 or more chronic illness (Table 1).

**Table 1.** Demographic Characteristics of 368 Participants at Baseline

Characteristic	Participants, No. (%)
Age, mean (SD), y	40.2 (5.9)
Weight at baseline, mean (SD), kg	84.0 (20.5)
Height, mean (SD), cm	170.4 (9.5)
Sex	
Female	209 (56.8)
Male	159 (43.2)
Weight status^a^	
Underweight	1 (0.3)
Normal weight	114 (31.0)
Overweight	124 (33.7)
Obese	129 (35.1)
Smoker	34 (9.2)
Aboriginal and Torres Strait Islander people	4 (1.1)
Born in Australia	279 (75.8)
Marital status	
Married or de facto	313 (85.1)
Separated, divorced, or widowed	29 (7.8)
Never married	26 (7.1)
No. of adults in household	
1	38 (10.3)
2	305 (82.9)
3	17 (4.6)
≥4	7 (1.9)
No. of children in household	
1	36 (9.8)
2	195 (53.0)
3	95 (25.8)
≥4	42 (11.5)
Educational level	
≤Year 10	17 (4.6)
Year 11-12	49 (13.3)
Certificate or diploma	125 (34.0)
University degree	177 (48.1)
Chronic illness	
None	163 (44.3)
1	107 (29.1)
>1	98 (26.6)
Annual weight change	
Stable (within 2.0%)	144 (45.7)
Loss (lost >2.0% of starting body weight)	84 (26.7)
Gain (gained >2.0% of starting body weight	87 (27.6)

^a^ Determined from baseline body mass index (calculated as weight in kilograms divided by height in meters square); patients were categorized as underweight (<18.5), normal weight (18.5-24.9), overweight (25.0-29.9), or obese (≥30.0).

### Weekly Weight Fluctuation

Across all participants and weeks of the year, detrended weight fluctuated by 0.3% each week (ie, for the mean [SD] weight of 84.0 [20.0] kg per participant, weight varied by 252 g each week). Results of the mixed-effects linear regression modeling for daily variation in weight (reference, Monday) are given in Table 2. Weights for all days of the week except Tuesday were significantly lower than weights on Monday. Thus, participants’ weight peaked on Mondays and Tuesdays (mean [SE] weight change, 0.01% [0.03%]; 95% CI, −0.05% to 0.06%; *P* = .83 for Tuesday) with weight gradually decreasing across subsequent weekdays (mean [SE] weight change, −0.08% [0.03%]; 95% CI, −0.13% to −0.03%; *P* = .003 for Wednesday; −0.15% [0.03%]; 95% CI, −0.21% to −0.10%; *P* < .001 for Thursday; and −0.18% [0.03%]; 95% CI, −0.24% to −0.13%; *P* < .001 for Friday) and increasing on the weekend (mean [SE] weight change, −0.16% [0.03]; 95% CI, −0.21% to −0.10%; *P* < .001 for Saturday and −0.10% [0.03%]; 95% CI, −0.15% to −0.05%; *P* < .001 for Sunday). When weekly weight fluctuation was considered separately based on annual weight change category, the amplitudes of weekly weight fluctuations were similar for the stable weight and weight gain groups, and relatively smaller for the weight loss group (eTable in Supplement 1).

**Table 2.** Daily Percentage Weight Change, Relative to Monday, for 368 Participants

Day of week	Mean (SE) weight change, % [95% CI]	*z* Score	*P* value
Monday [reference]	−0.13 (0.17) [−0.76 to 0.45]	NA	NA
Tuesday	0.01 (0.03) [−0.05 to 0.06]	0.21	.83
Wednesday	−0.08 (0.03) [−0.13 to −0.03]	−3.00	.003
Thursday	−0.15 (0.03) [−0.21 to −0.10]	−5.79	<.001
Friday	−0.18 (0.03) [−0.24 to −0.13]	−6.86	<.001
Saturday	−0.16 (0.03) [−0.21 to −0.10]	−5.77	<.001
Sunday	−0.10 (0.03) [−0.15 to −0.05]	−3.68	<.001

Abbreviation: NA, not applicable.

### Yearly Weight Change

**Figure 1.**
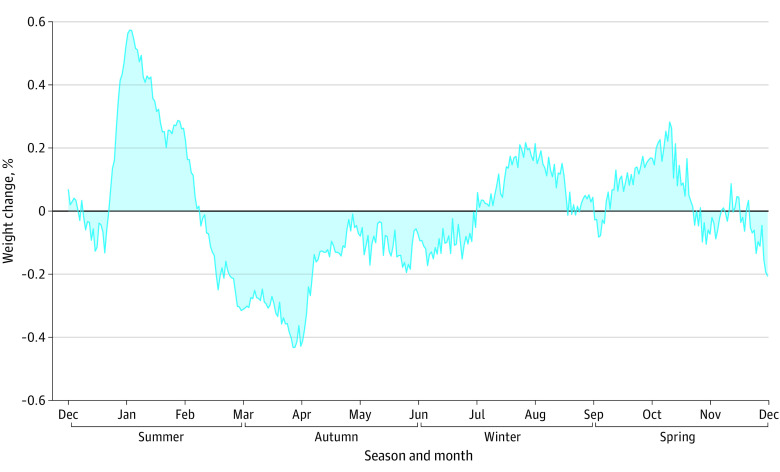
Yearly Weight Changes Among 368 Participants Data are based on mean percentage weight change.

Across the 12-month period, from December 1 to December 1 the following year, participants gained a median of 0.26% (218 g) body weight (mean [SE], –0.15%, [5.19%]; range, –29.4% to 24.0%). The overall annual pattern of weight change based on mean percentage daily weight change from baseline is presented in Figure 1. Visual observation of Figure 1 suggests that participants’ weight increased sharply during Christmas/New Year, gradually decreased from January to April (summer to autumn), slowly increased from April to October (autumn to spring), and then decreased from October to mid-December (spring to early summer). Mixed-effects linear regression modeling found that participants were heaviest in summer (significantly heavier than all other seasons); were lightest in autumn (mean [SE] weight change relative to summer, −0.47% [0.07%]; 95% CI, −0.59% to −0.34%; *P* < .001); regained some weight in winter (mean [SE] weight change relative to summer, −0.23% [0.07%]; 95% CI, −0.35% to −0.10%; *P* = .001); and were lighter in spring (mean [SE] weight change relative to summer, −0.27% [0.07%]; 95% CI, −0.40% to −0.14%; *P* < .001) (Table 3).

**Table 3.** Seasonal Change In Weight, Relative to Summer, for 351 Participants^a^

Season	Mean [SE] weight change [95% CI]	*z* Score	*P* value
Summer [reference]	0.05 (0.23) [−0.40 to 0.51]	NA	NA
Autumn	−0.47 (0.07) [−0.59 to −0.34]	−7.07	<.001
Winter	−0.23 (0.07) [−0.35 to −0.10]	−3.44	.001
Spring	−0.27 (0.07) [−0.40 to −0.14]	−4.08	<.001

Abbreviation: NA, not applicable.

^a^ Assessed using mixed-effects linear regression modeling.

Given that visual observation suggested a spike in weight at Christmas/New Year, mixed-effects linear regression analysis was used to compare weight in the week prior to the apparent spike (December 14 to 20) with the week following the spike (December 31 to January 6). The results suggested that weight increased by a mean of 0.65% (0.03%) (546 g) at Christmas/New Year (n = 361; *z* score, 25.30; *P* < .001).

Visual observation also suggested sharp weight gain in early April. Therefore, mixed-effects linear regression analysis was used to compare weight in the week prior to Good Friday to the week following Easter Monday (2020 wave: April 3 to 9 vs April 13 to 19; 2021 wave: March 26 to April 2 vs April 5 to 11). The results of the analysis suggested that weight significantly increase by a mean (SE) of 0.29% (0.02%) (244 g) at Easter (n = 342; *z* score, 11.51; *P* < .001).

### Yearly Weight Change by Weight Change Category

Annual weight change was considered separately for subgroups of 144 participants whose weight at 12 months was maintained within 2.0% of their starting body weight, 84 participants who lost more than 2.0% of their starting body weight, and 87 participants who gained more than 2.0% of their starting body weight.

**Figure 2.**
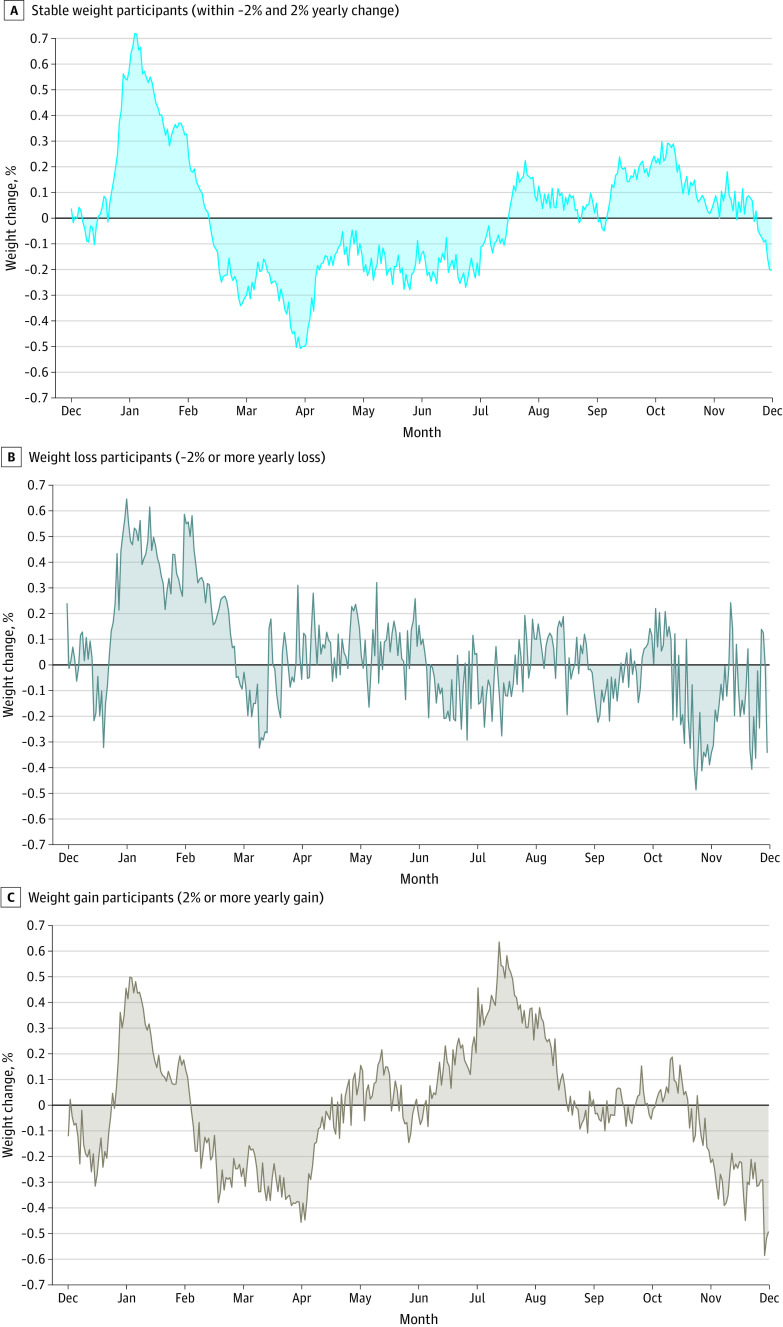
Yearly Weight Changes by 3 Weight Change Categories A, Includes 144 participants. B, Includes 84 participants. C, Includes 87 participants.

Detrended graphs by weight change subgroup were produced to facilitate visual inspection of weight gain or weight loss across the year (Figure 2). In all subgroups, a steep spike was seen in weight at Christmas/New Year of similar magnitude: 0.8% to 1.0% (range, 672 to 840 g). The annual weight fluctuation pattern for participants who maintained their weight was similar to that of the whole study sample, with gradual weight loss from January to April, followed by gradual weight gain from April to October, and a slight decrease from October to mid-December. In the group with weight gain, there was relatively higher winter weight gain, approximately 1.1% of weight, from April to July (924 g). By contrast, the weight loss group showed no winter weight gain.

## Discussion

This cohort study describes Australian adults’ weight changed across the year. Among participants, there was a median weight gain of 0.26% (218 g) across 12 months, although annual weight change varied widely. We identified cycles in weight on a weekly and seasonal basis and observed weight gain during the Christmas/New Year period. Participants tended to lose weight across the weekdays but increase weight on the weekends, with weight being lowest on Fridays. Weight was greatest in the southern hemisphere summer compared with all other seasons. Compared with summer, weight was lowest in autumn, followed by gradual weight gain in winter and early spring, followed by weight loss at the end of spring. Weight gain was greater in winter than in summer. There was a spike in weight gain at Christmas/New Year and Easter.

The weekly patterns we identified are consistent with the findings of Turicchi et al,^20^ who reported that weight was lowest on Fridays in 1061 to 1421 adults from UK, Denmark, and Portugal who were undergoing a weight-loss maintenance intervention. It is unclear whether these weekly variations are due to variation in energy intake or energy expenditure (or both) across the week, although a US study of 9000 adults found that weekend day energy intake was 6% higher than that on weekdays, with increased proportions of energy from fat and alcohol on weekends.^11^ Similarly, a study by An^21^ using National Health and Nutrition Examination Survey data of 11 646 adults between 2003 and 2012 found that energy intake was higher and diet quality was worse on the weekend than on weekdays. In addition, some studies have suggested physical activity is lower on weekend days than on weekdays.^22,23^ It is plausible that work schedules limit opportunities for overeating and excessive screen time.

In both the present study and in northern hemisphere–based studies,^24,25^ weight gain was greatest in winter. This underlying sinusoidal pattern of weight change (decreasing in summer, rising in winter) likely has a biological basis. Cold exposure appears to have an orexigenic effect in humans, as it does in other animals, which outweighs the increase in metabolic rate associated with increased thermogenesis, particularly for humans living in climate-controlled environments. Participants who gained weight across the 12-month study period had a strong pattern for wintertime weight gain, whereas participants who lost weight or whose weight was stable had little or no winter weight gain.

These weekly and seasonal patterns are overlaid with effects from holidays and festive seasons. Previous studies have also highlighted festive periods as being important for weight gain.^9^ A study by Turicchi et al^20^ found a spike in body weight at Christmas about twice as great as that in the present study. The somewhat more marked winter spike in the northern hemisphere is likely associated with the co-occurrence of Christmas and winter, whereas these 2 obesogenic factors are offset in the southern hemisphere.^24^ Alternatively, it is possible that the higher weight gain in the study by Turicchi et al^20^ was because their sample comprised people who had undergone a recent weight loss program.

Annual weight change in this cohort was modest. A mean annual increase of 282 g is equivalent to an energy excess of approximately 25 kJ/d, which would be imperceptible on either the expenditure or intake side of the energy balance equation (equivalent to approximately one-fifth of a square of chocolate or 1 minute of walking). However, there was wide variation, with 95% CIs for weight change (a loss or gain of approximately 10% of body weight), equating to very large deficits or excesses of 1750 kJ/d.

Change in weight is best understood by reference to a sociobiological model. Appetite increases and physical activity decreases in winter in both nonhibernating animals and in humans, carrying an obvious evolutionary advantage for homeotherms in times of food shortage. As a result, in environments where food is freely available, weight is likely to increase. In humans, this annual sinusoidal pattern is overlaid with complex patterns associated with social factors, including the weekly cycle of workdays and weekend days, annual holidays, and yearly festivities, which may influence, for example, the spikes in weight at Christmas/New Year and on weekends.

Weight loss and weight maintenance interventions may benefit from targeting temporal windows in which weight gain is known to occur. For Australian adults, there appear to be 2 key annual periods for weight gain: Christmas/New Year and winter. Furthermore, people who gain more weight across the year appear to be particularly susceptible during winter. Further research will be required to inform the development of appropriate components of any such interventions. In particular, it is unclear whether people would be receptive to a weight-gain prevention intervention at Christmas, given that it is traditionally viewed as a time of feasting and relaxing. Furthermore, the barriers needing to be addressed in a summer-time Christmas weight-gain prevention program would be quite different from those required for a winter-time weight-gain prevention program (such as exercising in inclement weather and shorter daylight hours).

It is interesting to contemplate whether there may be any health consequences of the semiannual weight cycle pattern detected in this study vs the annual cycle detected in previous studies conducted in northern hemisphere countries. Given that weight cycling is generally considered unfavorable, it seems possible that the more frequent cycling in Australians may be detrimental for health and may be contributing to the relatively high prevalence of overweight and obesity in Australia (for example, Australia has the fifth highest rate of obesity of 35 Organization for Economic Cooperation and Development countries^26^). In a similar vein, when annual weight change was graphed separately for subgroups of participants who maintained, lost, or gained weight in the present study (Figure 2), individuals who gained weight appeared to “yo-yo” by a greater amplitude across the year. Yo-yo patterns are associated with poor weight control^27^ and poorer health outcomes.^28,29^

Our study has some methodological implications for future research. First, it highlights the potential for using commercial smart devices combined with bespoke software to achieve low-burden, high-frequency data collection. Compliance was very good: participants weighed in a mean of 38 of 52 weeks in the year. There is considerable potential to harness this approach in the future to confirm and expand on the results of studies across other areas of Australia and regions of the world. Second, the seasonal weight cycling patterns suggest that the success of weight loss interventions may vary by season, and this should be considered in evaluating their effectiveness.

### Strengths and Limitations

This study has several strengths. To our knowledge, this is the first study to examine changes in weight across a full calendar year in any southern hemisphere country. This is of interest because of the different relationships between seasons and holidays and festivities in the southern hemisphere vs the northern hemisphere. A key strength of this study was its novel methodologic approach, which captured objectively measured weight at a high level of temporal resolution. The software developed for the study automatically harvested the weight data, removing the risk of data errors and reducing the risk of reactivity associated with using a participant logbook to collect weight data.

This study has limitations. We sampled adults from only 1 Australian city (Adelaide). Adelaide has a Mediterranean climate, with hot, dry summers and cool, wet winters. However, climates very widely across Australia, from tropical to alpine at the extremes, and it is unclear whether the results obtained in this study can be generalized to other climatic regions. Although participants were asked to weigh themselves as much as possible at the same time of day and in scant clothing, it is likely that variations in this protocol increased variability in weight measurements. Although reactivity was lower compared with the use of a manual logbook, the regular weighing protocol may have helped participants self-monitor their weight, which is a recognized weight maintenance strategy. However, the mean annual weight gain (282 g) observed in this study was much less than the 1 to 3 kg weight loss associated with frequent self-weighing in a systematic review by VanWormer et al^30^ and similar to the mean annual weight gain of 302 g for Australian adults aged 25 to 64 years who were followed up for 12 years in the Australian Diabetes, Obesity, And Lifestyle study.^5^ Finally, with a sample size of 368, it was not possible to conduct analyses for various subgroups.

## Conclusions

This cohort study examined changes in body weight of Australian adults and identified weekly, seasonal, and festive periods of weight gain. Results highlighted Christmas/New Year, which occur in summer in Australia, and winter as key periods for weight gain. Weight gain prevention interventions targeting those periods of the year appear warranted.

## Notes

Supplement 1. **eTable.** Mixed Model for Daily Variation in Weight

Supplement 2.
Data Sharing Statement
