# Supplementary material for: Weekly, Seasonal, and Festive Period Weight Gain Among Australian Adults
Source: JAMA Netw Open. 2023 Jul 27;6(7):e2326038. doi: 10.1001/jamanetworkopen.2023.26038 (PMC10375309; doi:10.1001/jamanetworkopen.2023.26038)
Supplement: Supplement 1. — eTable. Mixed Model for Daily Variation in Weight [file jamanetwopen-e2326038-s001.pdf]

Supplementary Online Content

Maher C, Ferguson T, Curtis R, et al. Weekly, seasonal, and festive period weight gain among Australian adults. *JAMA Netw Open*. 2023;6(7):e2326038.
doi:10.1001/jamanetworkopen.2023.26038

eTable. Mixed Model for Daily Variation in Weight

This supplementary material has been provided by the authors to give readers additional information about their work.

© 2023 Maher C et al. *JAMA Network Open*.

eTable. Mixed Model for Daily Variation in Weight. Mixed model for daily variation in weight (reference Monday) for participants whose: a) weight at 12 months was within 2% of their starting body weight (n=144); b) those who lost >2% of their starting body weight (n=84), and; c) those who gained >2% of their starting body weight (n=87).

Day of week	% Weight change	Standard error	Z score	p-value	95% confidence interval
Weight stable group (n=144)					
Monday (reference)	0.06	0.10	0.62	0.533	-0.13, 0.26
Tuesday	0.00	0.02	-0.19	0.851	-0.05, 0.04
Wednesday	-0.11	0.02	-4.40	0.000	-0.15, -0.06
Thursday	-0.19	0.02	-7.89	0.000	-0.24, -0.14
Friday	-0.23	0.02	-9.63	0.000	-0.28, -0.19
Saturday	-0.18	0.02	-7.41	0.000	-0.23, -0.13
Sunday	-0.10	0.02	-4.24	0.000	-0.15, -0.06
Weight gain group (n=87)					
Monday (reference)	2.87	0.22	13.06	0.000	2.44, 3.30
Tuesday	-0.04	0.06	-0.71	0.479	-0.15, 0.07
Wednesday	-0.14	0.06	-2.54	0.011	-0.26, -0.03
Thursday	-0.18	0.06	-3.21	0.001	-0.29, -0.07
Friday	-0.22	0.06	-3.78	0.000	-0.33, -0.10
Saturday	-0.17	0.06	-2.93	0.003	-0.28, -0.06
Sunday	-0.11	0.06	-1.85	0.064	-0.22, 0.01
Weight loss group (n=84)					
Monday (reference)	-3.42	0.40	-8.60	0.000	-4.20, -2.64
Tuesday	0.07	0.08	0.90	0.366	-0.08, 0.22
Wednesday	0.03	0.08	0.34	0.734	-0.12, 0.17
Thursday	-0.09	0.08	-1.22	0.223	-0.24, 0.06
Friday	-0.10	0.08	-1.37	0.172	-0.25, 0.04
Saturday	-0.13	0.08	-1.76	0.079	-0.28, 0.02
Sunday	-0.10	0.08	-1.33	0.183	-0.25, 0.05

© 2023 Maher C et al. *JAMA Network Open*.
