# Supplementary material for: Weekly, Seasonal, and Festive Period Weight Gain Among Australian Adults
Source: JAMA Netw Open. 2023 Jul 27;6(7):e2326038. doi: 10.1001/jamanetworkopen.2023.26038 (PMC10375309; doi:10.1001/jamanetworkopen.2023.26038)
Supplement: Supplement 2. — Data Sharing Statement [file jamanetwopen-e2326038-s002.pdf]

Data Sharing Statement

Maher. Weekly, Seasonal, and Festive Period Weight Gain Among Australian Adults. *JAMA Netw Open*. Published July 27, 2023. doi:10.1001/jamanetworkopen.2023.26038

Data
Data available: Yes
Data types: Deidentified participant data
How to access data: Upon reasonable request to the lead author, Carol Maher, at carol.maher@unias.edu.au
When available: With publication

Supporting Documents
Document types: None

Additional Information
Who can access the data: Researchers whose proposed use of the data has been approved
Types of analyses: For research purposes
Mechanisms of data availability: With investigator support, after approval of a proposal
Any additional restrictions: None
